# Linguistic markers for major depressive disorder: a cross-sectional study using an automated procedure

**DOI:** 10.3389/fpsyg.2024.1355734

**Published:** 2024-03-06

**Authors:** Raluca Nicoleta Trifu, Bogdan Nemeș, Dana Cristina Herta, Carolina Bodea-Hategan, Dorina Anca Talaș, Horia Coman

**Affiliations:** ^1^Department of Neurosciences, Discipline of Medical Psychology and Psychiatry, Iuliu Haţieganu University of Medicine and Pharmacy, Cluj-Napoca, Romania; ^2^Special Education Department, Faculty of Psychology and Education Sciences, Babeș-Bolyai University, Cluj-Napoca, Romania

**Keywords:** major depressive disorder, linguistic markers, LIWC, automated analysis procedure, cultural differences

## Abstract

**Introduction:**

The identification of language markers, referring to both form and content, for common mental health disorders such as major depressive disorder (MDD), can facilitate the development of innovative tools for early recognition and prevention. However, studies in this direction are only at the beginning and are difficult to implement due to linguistic variability and the influence of cultural contexts.

**Aim:**

This study aims to identify language markers specific to MDD through an automated analysis process based on RO-2015 LIWC (Linguistic Inquiry and Word Count).

**Materials and methods:**

A sample of 62 medicated patients with MDD and a sample of 43 controls were assessed. Each participant provided language samples that described something that was pleasant for them.

**Assessment tools:**

(1) Screening tests for MDD (MADRS and DASS-21); (2) Ro-LIWC2015 – Linguistic Inquiry and Word Count – a computerized text analysis software, validated for Romanian Language, that analyzes morphology, syntax and semantics of word use.

**Results:**

Depressive patients use different approaches in sentence structure, and communicate in short sentences. This requires multiple use of the punctuation mark period, which implicitly requires directive communication, limited in exchange of ideas. Also, participants from the sample with depression mostly use impersonal pronouns, first person pronoun in plural form – not singular, a limited number of prepositions and an increased number of conjunctions, auxiliary verbs, negations, verbs in the past tense, and much less in the present tense, increased use of words expressing negative affects, anxiety, with limited use of words indicating positive affects. The favorite topics of interest of patients with depression are leisure, time and money.

**Conclusion:**

Depressive patients use a significantly different language pattern than people without mood or behavioral disorders, both in form and content. These differences are sometimes associated with years of education and sex, and might also be explained by cultural differences.

## 1 Introduction

Major depressive disorder (MDD) is one of the most common diagnoses in mental health (WHO, 2023). Therefore, early identification of both new cases and relapses is a priority for both policy makers and clinicians, to aleviate its global burden.

The ICD-11 definition of depressive episode requires at least 5 out of 10 symptoms; one of the mandatory symptoms should be either depressed mood, or significantly diminished interest or pleasure in activities. Hopelessness regarding the future is not listed in DSM-5 as a symptom of major depression (DSM, 2013), nevertheless the ICD-11 includes it due to its power to discriminate patients with depression from those without (WHO, 2022). Other depression symptoms involving cognition include thoughts of low self-worth, guilt, one’s own death, suicide. Among depression symptoms relevant for cognitive functioning, ICD-11 also lists decreased sustained attention and concentration, and significant indecisiveness. The symptoms should be present for at least 2 weeks, almost daily and most of the time in the occurring days; they should not be secondary to another health condition, medication or substance use, or bereavement. Moreover, they should generate significant functional impairment.

Building on these descriptors, existing studies explore the relationship between language and depression, as language is a natural way through which we elicit an outward expression of thoughts, emotions and other mental processes. The challenge resides in our ability to identify language features that convey information about interest, pleasure, self-confidence, indecision, self-esteem, hopelessness, diminished appetite, social withdrawal and other main features of depression in a form that can be used by natural language processing (NLP) machines.

### 1.1 Language as a biomarker for depression

Literature suggests that depression influences how individuals communicate, and multiple studies are performed in this direction. Recent research (Koops et al., 2023) states that depressive speech is characterized by several anomalies, such as lower speech rate, less pitch variability and more self-referential speech; moreover, data shows that current technologies are able to predict these features in depression with an accuracy of up to 91%. Our previous studies (Trifu et al., 2015, 2017) aimed to explore the potential of language markers in major depressive disorder (MDD) via manual analysis and identified specific patterns of language, specific depression language markers, and their relationships with cognitive functioning.

Recent studies brought increasingly conclusive results that support the hypothesis of mood disorders imprint on language. Linguistic imprints of mood disorders appear in every component of language, more specifically the lexical, semantic, morphological unit, syntax, pragmatic and social communication, respectively. Language assessment becomes more accessible if broken down into the aforementioned components.

Regarding language lexicology and morphology, a study of two bilingual samples identified the following linguistic imprints: self-referential language, 1st person speech using 1st person singular pronoun (‘I’), increased amount of negative emotion words with decreased use of positive emotion words, and decreased use of 1st person plural speech (‘we’), respectively (Behdarvandirad and Karami, 2022). Another study reports similar results for the use of negative emotion and 1st-person singular. Moreover, the sample of participants with depression more often used words connected with work, family, sex, biology, and health. Additionally, the use of past tense, causation, achievement, family, death, psychology, impersonal pronouns, quantifiers, and preposition words outlined emotion-dependent differences between the sample with depression and controls (Yang et al., 2023). Another research supports these findings with similar results (Robertson et al., 2023).

Persons with depression also use fewer future tense constructions (e.g., will), fewer high-certainty constructions (certainly), more low-certainty constructions (could) and more deontic, especially volitive boulomaic modal constructions, such as hopes, wishes and desires. Also, absolute meaning words are more frequent in depression in general (Al-Mosaiwi and Johnstone, 2018; Yahya and Rahim, 2023). This concurs with the cognitive rigidity theory in depression and mental health (Aguilera et al., 2019).

Diminished communication in persons with depression reflects in, and is altered by, the language syntax. The language displayed by a person with depression indicates use of truncated sentences, with omissions, short sentences and reversed topic of the sentence, which in turn render the message of communication difficult to understand (Trifu et al., 2017). Single-clause sentence predominance over multi-clause and atypical word order is common in the group of depressive persons. Modified word order, presence of ellipses and colloquialisms were the highest predictors in discriminating between persons with and without depression (Smirnova et al., 2018). Similarly, simple sentences vs. complex syntax can predict successful outcomes of recovery programs; more specifically, complex syntax predicts wellbeing (Zinken et al., 2010).

Regarding the pragmatic component of the language, more precisely the use of natural language, colloquial expressions, and idioms that facilitate interpersonal communication and social relations, a recent study (Bridges et al., 2023) shows specific patterns of language. Based on language analysis related to naturalistic/ formulaic expressions in patients with treatment-resistant depression who have benefited from surgery, i.e., surgical deep brain stimulation of the subcallosal cingulate white matter pathways, the study indicates that patients with depression produced fewer conversational speech expressions pre- and post-operatively, compared with healthy controls. The study also ascertained a higher rate of non-nuanced familiar expressions, large lexical bundles, e.g., “it is used to,” “on the other hand,” “finally, whatever” that are fixed linguistic segments. In this respect, studies ascertain (Biber, 2004; Zhang et al., 2021) that lexical bundles create building blocks of discourse and actively contribute to fluent linguistic production; however, they reduce interactivity in communication, due to their stereotypical nature. This contrasts with the decreased use of nuanced expressions, formulaic expressions post-operatively, e.g., “Er,” “Uhm,” “Oh” and conversational speech formulas, e.g., “Are you ok? “You’ve got to be kidding,” “Excuse me?,” which elicits more personal communication and subsequently better interpersonal relationships.

### 1.2 Computational analytic approaches to language in depression

In recent years, given the widespread use of social media and access to technologies, text analysis has emerged as a promising way to gather information about individuals and their illnesses. A comparison between human raters and NLP machines, such as Linguistic Inquiry and Word Count (LIWC) was performed in the medical field regarding self-reported psychological and physical health, based on written essays of chronic pain patients. The authors (Ziemer and Korkmaz, 2017) ascertained a better predictive power for human raters than computerized text analysis on measures of depression, but LIWC and human raters had similar predictive power in other medical variables, such as pain severity, pain catastrophizing and illness intrusiveness. Nevertheless, other studies (Burkhardt et al., 2021; Dudău and Sava, 2022; Spruit et al., 2022; Santos et al., 2023) proved the efficacy of LIWC in identifying depressive mood and other mental health indicators from language. Burkhardt monitored in his longitudinal research the linguistic indicators in persons with depression and underlined that LIWC markers of depression and novel linguistic indicators of activation, such as linguistic indicators of planning and participation in enjoyable activities, strongly associate with depression scores, evaluated with the Patient Health Questionnaire 9 (PHQ-9), and longitudinal patient trajectories, respectively. Likewise, emotional tone, pronoun rates, words related to sadness, health, and biology, and behavior activation-related LIWC categories, respectively, appear to be complementary. Another study (Coello-Guilarte et al., 2019) managed to reasonably identify depression with LIWC even in the cross-linguistic context, i.e., by using an automatic translation of texts and bilingual dictionaries. Kimball et al. (2019) proved that LIWC is a sensitive tool in screening for anxiety and depression in tinnitus patients, even when the self-assessment fails to indicate relevant levels of anxiety and depression symptoms. Furthermore, a longitudinal study in which LIWC assessment combined with other Coefficient for Naturalistic Language processing (NLP) tools such as SentiWordNet, LDA Topic and Word2Vec, indicated that language processing and analysis moderately predicts depression risk onset among pregnant persons, 30 days and 60 days after giving birth, respectively (Krishnamurti et al., 2022).

Thus, we may state that the study of the relationship between language and depressive disorder is a current, highly interesting topic. However, studies in this direction are only at the beginning and are difficult to implement due to linguistic variability, the difficulty of qualitative analysis of linguistic samples, the specificity of language, cultural context in relationship with language and depressive disorder, and the many different approaches to language evaluation used until now.

## 2 Research goals and hypotheses

### 2.1 Study relevance

The current, cross-sectional study uses an automated procedure and performs a computerized comparative assessment of language markers through the Ro-LIWC 2015 in a clinical sample of MDD participants and a control sample. Based on our knowledge, and the literature search, this is the first study that applies the LIWC- RO 2015 in a clinical sample with depression. Previous validation studies (Dudău and Sava, 2022) use depressive language from books and literature, not clinical samples. Moreover, the current study complements the LIWC - 2015-RO validation study, one of the limits of Dudau’s study being the lack of clinical samples. Furthermore, many of the previous studies used screening instruments like the PHQ-9 in the evaluation of depressive symptoms. We set out to use the Montgomery-Asberg Depression Rating Scale (MADRS) instead, which is a clinician-rated instrument designed specifically for the sensitive evaluation of the intensity of depressive symptoms (Montgomery and Asberg, 1979).

The current study provides relevant, valuable data regarding linguistic markers for Romanian language and cultural context, since both the clinical and control samples who underwent automated assessment and rating are Romanian. Furthermore, such findings can be useful in building pre-trained language based models to augment feature-based dictionaries for programming machines to identify people at risk for or suffering from depression (Rawsthorne et al., 2020; Balagopalan et al., 2021; Malins et al., 2022; Yu et al., 2022).

### 2.2 Materials and methods

#### 2.2.1 Participants

We included a sample of 62 participants with clinical depression, diagnosed with MDD based on ICD-10 criteria by two independent clinicians, and a sample of 43 controls without any history of mental health illness or mood disorders, assessed on ICD-10 criteria by two independent clinicians. The sample with depression consisted of psychiatric inpatients from Cluj County Emergency Hospital and outpatients under psychiatric medication. Exclusion criteria were: age (less than 18, more than 65) and the existence of other psychiatric disorders, confirmed also by the clinicians. Inclusion criteria for the control group were: age (between 18 and 65), no history of psychiatric disorder, and no current psychiatric symptoms, confirmed by two independent clinicians. Each participant was evaluated with clinical tests and language tests, provided language samples and described something that made them happy or was pleasant for them.

#### 2.2.2 Assessment tools

1. Depression, Anxiety and Stress Scale 21 (DASS-21) is a 21 item clinical instrument that assesses three dimensions of the emotional state present during the previous week (Lovibond et al., 2011). It is a widely used scale, that has been translated, adapted and validated in numerous languages, including Romanian.
2. The Montgomery-Asberg Depression Rating Scale (MADRS) is one of the most widely used clinical instruments for quantifying the severity of depressive symptoms. It is a 10 item scale that covers the main characteristics of depression; it has the advantage of being more specific for depression than other scales since it does not include items that can be associated with other mental disorders (e.g., anxiety), and it is very sensitive to change (Montgomery and Asberg, 1979).
3. Linguistic Inquiry and Word Count (Chung and Pennebaker, 2013, 2018; Pennebaker et al., 2015) is a closed vocabulary approach, a tool that allows researchers to analyze specific language data. More precisely, it consists of an internal dictionary and an automated software design for language classification and word count. The instrument was developed initially in English and, with the help of technology and dictionaries, it was adapted in other languages. Easy download and use, affordability and wider range of content and grammar features (Chung and Pennebaker, 2018), make it an ideal candidate for analyzing written language and language samples. Currently, the LIWC 2015 website (Pennebaker, 2023) lists the tool in 22 available languages, including Romanian.

LIWC became a preferred research tool for language analysis. In 2023, a simple search in the WOS Core Collection databases with the “topic” search key criteria indicated a number of 657 researches and 667 for “all fields” search key criteria. Some of these studies are validation and adaptation studies, but some are applicative and highlight the use of LIWC in different contexts of language analysis. LIWC 2015 “all fields” search key criteria indicates 54 studies, 16 of which with specific focus on LIWC-2015. From these, 7 are of interest for health and education (Stanton et al., 2017; Chippendale and Gentile, 2021; Monzani et al., 2021; Ansari and Du, 2022; Yang et al., 2022; Sengun et al., 2023; Wu et al., 2023). Moreover, 7 studies (Toma and D’Angelo, 2015; Coello-Guilarte et al., 2019; Kimball et al., 2019; Berkout et al., 2020; McDonnell et al., 2020; Dudău and Sava, 2022; Meyerhoff et al., 2023) focus on the use of LIWC in connection with depression, all of them relevant for the topic of this study. The value of LIWC in mental health and depression studies stems from its perspective on software analysis. More specifically, LIWC distinguishes between content categories and functional categories; furthermore, it identifies different hierarchical patterns of structuring of the 2 aforementioned categories.

LIWC 2015 has been validated for Romanian Language (Ro-LIWC 2015) by Dudău and Sava (2021, 2022). For the current study, the analyzed categories were: (a) Word function: articles, prepositions, auxiliary verbs, adverbs, conjunctions, negations; (b) Other grammar: verbs, adjectives, comparisons, interrogatives, numbers; (c) Affect words: positive, negative, anxiety, anger, sadness; (d) Social words: family, friends, female, male; (e) Cognitive process: insight, causations, discrepancy, tentative, certainty, differences; (f) Perceptual processes – see, hear, feel; (g) Biological processes: body, health, sexual; (h) Drives: affiliation, achievement, power, reward, risk; (i) Time oriented: past, present, future; (j) Personal concern: work, leisure, home, money, religion, death; (i) Informal language: swear, net speak, agreement, non – fluencies, filler words.

#### 2.2.3 Procedure

We used interviews for collecting language samples. Each participant provided approximately 5 min of narrative production. The participants were asked to talk about something that is pleasurable for them. As language occurs in a natural manner, we used open–ended personal narrative questions. The investigator request was:” Please describe something that you like. What are your hobbies?.” Based on the depressive condition, the investigator rephrased with:” Please describe something that you enjoyed doing before you experienced this illness? Did you have any hobby?.” Speech recording was performed with a Sony Recorder. Cloud Speech API was used to transcript from audio.mp3 and audio.wav to text files, followed by manual editing, transcripts and verification, using WavePad Sound Editor and the text editor. Subsequently, the conversations were converted into Microsoft Ofice Excell Pack, and the data were exported for analysis in LIWC- Ro2015, which generated the data used for analysis.

The study was approved by the Ethical Committee of the Iuliu Hatieganu University of Medicine and Pharmacy Cluj Napoca, with the number Av. 227/7.02.2022.

#### 2.2.4 Statistical analysis

Data analysis was performed in R version 4.3.1. Normally distributed data is presented as mean ± standard deviation. Non-normally distributed data is presented as median (1st quartile; 3rd quartile). The Chi-square test was used to analyze differences in qualitative variables distribution across groups. The Wilcoxon Rank Sum test (Mann–Whitney U test) was used to assess the differences between non-normally distributed variables across groups. The Kolmogorov–Smirnov test was used to assess the normality of data distribution. Regression models were used to control for the effect of sex, age, and years of education on the significant differences in language parameters across groups. The models were built using a step-by-step approach, where we first tested for collinearity between age and years of education, then we introduced, consecutively, the variables group, sex, and then years of education in the regression model, if relevant for each language parameter. At each step, the standard error of the coefficient of interest was monitored for significant increase (suggestive for high collinearity). Variables that generated high collinearity were excluded from subsequent models. Spearman and Pearson correlation coefficients were calculated, according to data distribution, to test for linearity.

## 3 Results

The demographic characteristics of the two samples are presented in Table 1. Depressive patients were significantly older than controls and had significantly lower years of education.

**Table 1:** Demographic characteristics of study sample.

	Patients	Controls	*p*
N	62	43	
Sex (% females)	68.25%	78.38%	NS 1
Age (years)	54.0 (46.5; 58.0)	35.0 (30.0; 39.0)	< 0.001 2
Education (years)	12.0 (10.0; 13.0)	16.0 (16.0; 16.5)	< 0.001 2

1 Chi-squared test; 2 Wilcoxon rank sum test (Mann–Whitney *U* test).

Concerning the presence of depressive symptoms, the sample with depression recorded on DASS–21 a score of M = 10 (7; 15) and Controls recorded a score of M = 1 (0; 3); concerning the severity of depression measured with MADRS, the sample with depression scored M = 31 (25; 36), compared with Controls M = 2 (0; 4).

The differences in language parameters between depressive patients and controls are summarized in Table 2.

**Table 2:** Differences in low-level features between groups.

	Patients	Controls	*p*
	(*N* = 62)	(*N* = 43)	
Word count	347.0 (195.0; 492.5)	376.0 (246.0; 486.0)	NS ^1^
Words per sentence	11.47 (8.49; 14.98)	22.11 (16.36; 27.00)	< 0.001 ^1^
Big words	12.71 (10.93; 14.90)	14.66 (12.35; 16.46)	0.021 ^1^
Dictionary	78.51 (76.19; 81.00)	79.09 (76.85; 81.07)	NS ^1^
Function	47.91 (43.78; 50.33)	47.24 (44.14; 49.29)	NS ^1^
Pronoun	12.10 (10.29; 14.05)	13.62 (11.38; 14.75)	NS ^1^
Personal pronouns	9.52 (7.74; 11.35)	9.92 (8.11; 11.85)	NS ^1^
I	5.15 (3.87; 6.91)	5.59 (3.69; 6.76)	NS ^1^
We	0.14 (0.00; 0.59)	0.54 (0.00; 0.69)	0.019 ^1^
You	0.28 (0.00; 0.72)	0.33 (0.00; 0.81)	NS ^1^
She / He	2.29 (1.64; 3.12)	2.37 (1.80; 3.40)	NS ^1^
They	0.76 (0.38; 1.48)	0.95 (0.52; 1.54)	NS ^1^
Impersonal	2.52 (1.59; 3.16)	3.02 (2.16; 4.14)	0.024 ^1^
Other function words	-	-	-
Articles	2.01 (1.52; 2.84)	2.01 (1.39; 2.86)	NS ^1^
Prepositions	9.40 (8.15; 11.13)	10.90 (9.35; 12.09)	0.009 ^1^
Auxiliary verbs	7.87 (5.66; 9.69)	4.65 (2.43; 8.24)	< 0.001 ^1^
Adverbs	11.72 (10.00; 13.59)	10.91 (9.11; 13.41)	NS ^1^
Conjunctions	8.89 (6.90; 10.13)	7.58 (5.75; 8.88)	0.010 ^1^
Negations	5.34 (3.60; 6.64)	3.71 (2.44; 5.54)	0.006 ^1^
Other grammar	-	-	-
Verbs	23.08 (21.12; 25.40)	22.32 (19.90; 23.60)	NS ^1^
Adjectives	5.97 (4.31; 7.37)	5.77 (4.53; 7.83)	NS ^1^
Comparisons	2.53 (1.68; 3.97)	3.38 (2.22; 4.52)	NS ^1^
Interrogatives	2.64 (1.64; 3.28)	3.53 (2.41; 3.92)	0.006 ^1^
Numbers	2.06 (1.42; 3.13)	2.13 (1.72; 2.76)	NS ^1^
Quantifiers	2.17 (1.37; 3.16)	2.25 (1.55; 3.53)	NS ^1^
Affect	6.81 (5.15; 7.81)	7.51 (5.71; 9.41)	NS
Positive	3.48 (2.47; 5.16)	5.66 (3.99; 7.01)	< 0.001
Negative	2.40 (1.42; 3.47)	1.54 (1.01; 2.35)	0.011
Anxiety	0.29 (0.00; 0.68)	0.00 (0.00; 0.25)	0.007
Anger	0.34 (0.00; 0.69)	0.31 (0.00; 0.61)	NS^1^
Sadness	0.76 (0.14; 1.57)	0.38 (0.00; 1.03)	NS^1^
Social	7.44 (5.67; 9.04)	7.00 (5.66; 8.78)	NS
Family	0.51 (0.23; 1.06)	0.27 (0.00: 0.92)	NS^1^
Friend	0.00 (0.00; 0.26)	0.00 (0.00; 0.27)	NS^1^
Female	0.63 (0.00; 1.06)	0.48 (0.00; 0.80)	NS^1^
Male	0.87 (0.43; 1.28)	0.47 (0.00; 1.50)	NS^1^
Cognitive processes	17.62 (15.62; 21.84)	19.56 (16.37; 21. 20)	NS^1^
Insight	1.98 (1.20; 3.58)	2.86 (1.65; 4.12)	NS^1^
Causation	3.96 (2.82; 5.01)	3.23 (2.35; 4.39)	NS^1^
Discrepancy	4.35 (3.08; 5.64)	3.55 (2.35; 4.72)	NS^1^
Tentative	4.72 (2.83; 6.10)	5.30 (4.27; 7.79)	0.045
Certainty	1.84 (1.48; 2.55)	2.07 (1.53; 2.35)	NS^1^
Difference	5.76 (4.32; 7.00)	5.00 (3.76: 5.79)	NS^1^
Perceptual processes	2.67 (1.75; 3.52)	2.78 (2.21; 4.16)	NS^1^
See	0.91 (0.33; 1.64)	0.81 (0.38; 1.94)	NS^1^
Hear	0.84 (0.27; 1.48)	0.74 (0.27; 1.15)	NS^1^
Feel	0.55 (0.06; 1.06)	0.60 (0.20; 0.83)	NS^1^
Biological processes	2.63 (1.63; 3.55)	1.42 (0.77; 1.89)	< 0.001
Body	0.54 (0.08; 1.02)	0.32 (0.00; 0.93)	NS^1^
Health	0.88 (0.52; 1.49)	0.25 (0.00; 0.57)	<0.001
Sexual	0.00 (0.00; 0.00)	0.00 (0.00; 0.00)	NS^1^
Ingest	0.58 (0.00; 1.49)	0.16 (0.00; 0.55)	0.013
Drives	10.00 (8.51; 11.91)	8.31 (6.72; 9.96)	0.010
Affiliation	0.77 (0.36; 1.40)	1.49 (1.01; 2.13)	0.002
Achievement	3.94 (2.62; 5.26)	2.77 (2.13; 3.87)	0.013
Power	3.21 (2.38; 4.24)	2.30 (1.63; 3.45)	0.009
Reward	0.95 (0.31; 1.64)	1.15 (0.70; 1.55)	NS^1^
Risk	1.52 (0.99; 2.39)	1.27 (0.62; 1.86)	NS^1^
Time orientation	-	-	-
Past	10.79 (7.83; 13.74)	6. 47 (4.61; 9.71)	<0.001
Present	14.66 (11. 99; 17.05)	16.06 (13.96; 17.42)	NS^1^
Future	0.61 (0.00; 1.18)	0.48 (0.00; 0.90)	NS^1^
Relativity	17.20 (14.35; 19.27)	16.22 (13.38; 20.00)	NS ^1^
Motion	3.39 (2.25; 4.88)	3.75 (3.02; 4.72)	NS ^1^
Space	8.64 (6.65; 9.62)	8.04 (6.51; 10.59)	NS ^1^
Time	6.90 (5.29; 7.87)	4.92 (3.49; 7.17)	0.035 ^1^
Personal concerns	-	-	-
Work	1.92 (0.98; 3.29)	1.38 (0.63; 2.39)	NS ^1^
Leisure	1.09 (0.59; 1.84)	2.21 (1.51; 3.52)	< 0.001 ^1^
Home	0.55 (0.00; 0.95)	0.22 (0.00; 0.62)	0.013 ^1^
Money	0.27 (0.00; 0.65)	0.00 (0.00; 0.21)	0.003 ^1^
Religion	0.00 (0.00; 0.23)	0.00 (0.00; 0.18)	NS ^1^
Death	0.00 (0.00; 0.14)	0.00 (0.00; 0.00)	NS ^1^
Informal language	1.09 (0.66; 1.93)	0.92 (0.38; 1.42)	NS ^1^
Swear	0.00 (0.00; 0.00)	0.00 (0.00; 0.00)	NS ^1^
Net speak	0.00 (0.00; 0.18)	0.00 (0.00; 0.23)	NS ^1^
Agreement	0.90 (0.44; 1.79)	0.57 (0.31; 1.04)	0.027 ^1^
Non-fluencies	0.00 (0.00; 0.00)	0.00 (0.00; 0.00)	NS ^1^
Filler words	0.00 (0.00; 0.00)	0.00 (0.00; 0.00)	NS ^1^
All punctuation	32.12 (24.95; 41.83)	22.95 (19.75; 26.35)	< 0.001 ^1^
Period	21.37 (12.99; 30.40)	12.76 (10.11; 16.36)	< 0.001 ^1^
Comma	6.84 (4.64; 8.36)	7.28 (5.59; 10.00)	NS ^1^
Question mark	0.00 (0.00; 0.29)	0.16 (0.00; 0.31)	NS ^1^

* Controlled for sex and years of education; NS, non-significant (*p* > 0.05); 1 Wilcoxon Rank Sum test (Mann–Whitney *U* test).

**Figure 1:**
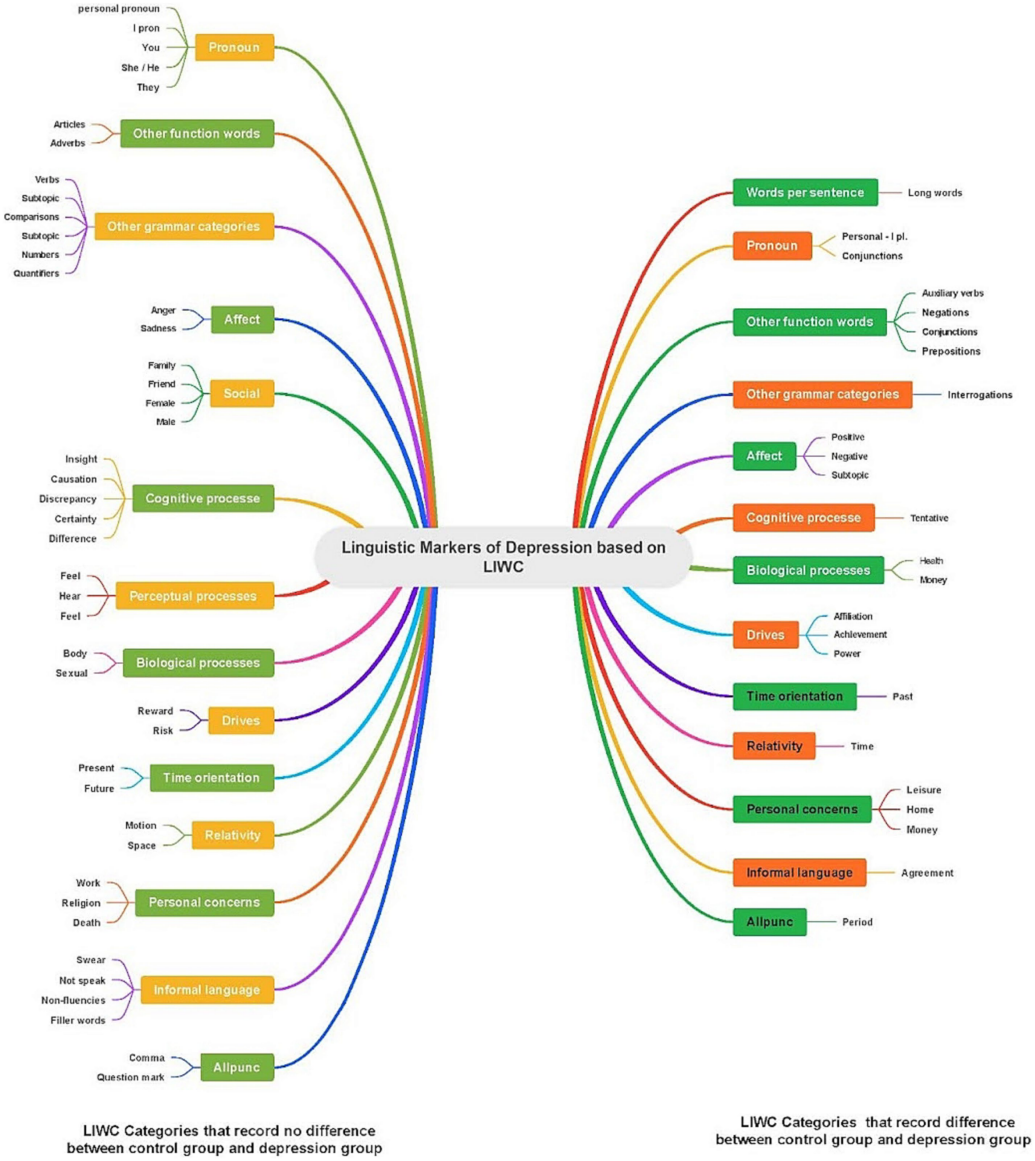
The difference based on LIWC categories between the sample with depression and controls.

The overview of differences regarding LIWC between the sample with depression and controls is presented in the Figure 1. The significant differences recorded refer to all aspects of language, i.e., morphology, syntax and semantics.

Age and years of education were significantly correlated (Spearman’s rho = −0.544 – *p* < 0.001) in our samples. Therefore, to avoid breaking the collinearity assumption, we only included sex and years of education in the regression models. The relationships between language parameters that were significantly different between our samples on the one side, and sex and years of education on the other side, are summarized in Table 3.

**Table 3:** Relationship between significantly different language parameters across groups and sex and years of education.

	Sex			Years of education	
	Males	Females	*p*	Spearman’s rho	*p*
Words Per Sentence	12.08	15.29	0.011^1^	0.535	< 0.001
	(8.85; 15.97)	(11.38; 22.37)			
Big words	13.03	13.45	NS^1^	0.366	< 0.001
	(11.07; 14.74)	(11.19; 15.88)			
Pronoun	-	-	-	-	-
We	0.27	0.29	NS^1^	0.162	NS
	(0.00; 0.86)	(0.00; 0.69)			
Impersonal	2.67	2.65	NS^1^	0.168	NS
	(1.67; 3.09)	(1.89; 3.71)			
Other function words	-	-	-	-	-
Prepositions	9.29	10.32	NS^1^	0.306	0.003
	(8.10; 11.46)	(8.65; 11.84)			
Auxiliary verbs	8.17	6.24	NS^1^	−0.248	0.017
	(6.39; 9.11)	(3.86; 9.35)			
Conjunctions	8.35	8.33	NS^1^	−0.185	NS
	(6.79; 9.49)	(6.17; 9.97)			
Negations	5.30	4.63	0.025^1^	−0.277	0.007
	(4.13; 6.92)	(2.72; 5.92)			
Other grammar	-	-	-	-	-
Interrogatives	3.12	2.69	NS^1^	0.106	NS
	(2.43; 3.62)	(1.62; 3.72)			
Affect	-	-	-	-	-
Positive	3.47	4.57	NS^1^	0.339	< 0.001
	(2.90; 5.16)	(2.93; 5.87)			
Negative	2.08	1.99	NS^1^	−0.299	0.004
	(1.47; 3.22)	(1.18; 3.16)			
Anxiety	0.00	0.20	NS^1^	−0.269	0.009
	(0.00; 0.38)	(0.00; 0.54)			
Cognitive processes	-	-	-	-	-
Tentative	5.27	4.83	NS^1^	0.205	0.048
	(3.06; 6.88)	(3.16; 6.16)			
Biological processes	2.06	1.99	NS^1^	−0.370	< 0.001
	(0.97; 3.51)	(1.21; 3.14)			
Health	0.80	0.62	NS^1^	-0.312	0.002
	(0.14; 1.30)	(0.22; 1.00)			
Ingest	0.48	0.41	NS^1^	-0.359	<0.001
	(0.00; 0.86)	(0.00; 1.30)			
Drives	10.64	8.77	0.009^1^	-0.287	0.005
	(9.03; 12.65)	(7.32; 10.90)			
Affiliation	0.94	1.12	NS^1^	0.271	0.009
	(0.53; 1.70)	(0.51; 1.57)			
Achievement	3.73	3.20	NS^1^	-0.324	0.002
	(2.85; 4.73)	(2.16; 4.91)			
Power	3.24	2.87	NS^1^	-0.319	0.002
	(2.50; 4.09)	(1.64; 3.83)			
Time orientation	-	-	-	-	-
Past	10.97	8.52	NS^1^	-0.310	0.002
	(8.17; 12.36)	(5.65; 12.84)			
Relativity	-	-	-	-	-
Time	6.77	6.37	NS^1^	-0.187	NS
	(4.73; 8.24)	(4.40; 7.71)			
Personal concerns	-	-	-	-	-
Leisure	1.78	1.38	NS^1^	0.353	0.001
	(0.69; 3.21)	(0.85; 2.43)			
Home	0.25	0.51	NS^1^	-0.262	0.011
	(0.00; 0.93)	(0.00; 0.85)			
Money	0.00	0.19	NS^1^	-0.248	0.017
	(0.00; 0.55)	(0.00; 0.51)			
Informal language	-	-	-	-	-
Agreement	0.79	0.73	NS^1^	-0.065	NS
	(0.50; 1.76)	(0.34; 1.29)			
All punctuation	32.80	25.34	0.005^1^	-0.488	< 0.001
	(25.93; 43.01)	(22.06; 30.97)			
Period	21.45	14.31	0.004^1^	-0.426	< 0.001
	(16.91; 29.46)	(10.86; 21.82)			

NS = non-significant (*p* > 0.05); 1 Wilcoxon rank sum test (Mann–Whitney *U* test).

The best regression models (i.e., highest multiple R2, no parameter without statistical significance, and no highly collinear variables) built for controlling for the influence of sex and years of education are shown in Table 4. Regression models in which adding sex or years of education did not improve the model are not shown, i.e., models built to control for the confounding effect of sex and years of education on: prepositions, auxiliary verbs, negations, positive emotions, negative emotions, anxiety, tentative, biological processes, health, ingest, affiliation, power, focus on the past, leisure, home, and money. In all models, the sample retained an (independent) effect, except for Big Words and achievement, for which years of education was a better predictor.

**Table 4:** Regression models.

Item	Intercept		Group		Sex		Education		mR^2^	F	p
	b	SE	b	SE	b	SE	b	SE			
Words Per Sentence	14.62^***^	5.10	−7.95^***^	1.84	3.30^*^	1.37	-	-	0.38	30.26	< 0.001
Big words	9.32^***^	1.50	-	-	-	-	0.32^**^	0.11	0.09	9.19	0.003
Drives	10.08^***^	0.77	1.44^*^	0.67	−1.90^**^	0.72	-	-	0.12	6.51	0.002
Achievement	6.69^***^	1.61	-	-	-	-	−0.21*	0.09	0.10	5.11	0.008
Allpunc	27.98^***^	2.24	10.51^***^	1.94	−6.62^**^	2.09	-	-	0.31	21.78	< 0.001
Period	18.28^***^	2.39	9.30^***^	2.07	−6.27^**^	2.23	-	-	0.24	15.62	< 0.001

mR^2^, Multiple R-squared; F, F-statistic; *, significant at the 0.05 level; **, significant at the 0.01 level; ***, significant at the 0.001 level.

## 4 Discussion

The current study investigates the linguistic markers and changes in narrative language in a sample of patients with depression versus controls. The study used an automated speech analysis, the Romanian version of LIWC 2015, for the assessment. Our study shows specific patterns of language in the sample of patients with depression, who display specific language markers. Nevertheless, we identified slight differences from our previous studies.

Regarding the choice of the instrument, the hierarchical structure of LIWC is significantly more relevant and brings a better outlook on the relationship between thinking and language, compared with other types of linguistic content analysis, manual or automatized, such as SentiWordNet, SentiStrenght, ANEW or General Inquire see (Dudău and Sava, 2022), SetembroBR corpus (Santos et al., 2023), Inflexitext (Berkout et al., 2020). For instance, the Affect category in LIWC is a superordinate category, defined by several lower order categories (subcategories) listed under the aforementioned category, more specifically Positive Affect, Negative Affect, Anxiety, Anger, Sadness. Thus, LIWC may generate significant information from the relationship between the category, i.e., affect, and another psychological construct, such as a cognitive process, or with one or more of the five aforementioned lower order categories, respectively. Moreover, the hierarchical sub-category of 1st person pronoun use in LIWC, listed in the superordinate category of Pronouns, appears significantly important for the diagnosis of depression. This result emerges from a meta-analysis (Edwards and Holtzman, 2017) focused on the association between depression and the use of first-person singular pronouns in a sample of *k* = 21, *N* = 3.758, using LIWC. The results revealed a small correlation *r* = 0.13, 95% CI = [0.10–0.16] and the authors conclude that first-person singular pronouns can be used as a linguistic marker of depression, in a manner transcending demographic parameters and not mediated by gender. The first-person pronoun is also discussed when addressing how interpersonal connections are reflected in the relationship between language and depression (Meyerhoff et al., 2023). People with higher levels of depressive symptoms (PHQ-8) tend to use more differentiation words; (1) connected with close contacts, they used more first-person singular, filler, sexual, anger, and negative emotion words; (2) connected with non-close contacts, they used more conjunctions, tentative, and sadness-related words, and fewer first-person plural constructions. A study carried out by Smirnova et al. (2018) showed slight differences between the samples with depression and normal sadness, respectively. The use of reflexive (e.g., myself) vs. personal pronouns is different in patients with mild depression. This concurs with the initial results of a study that compared the written language of students with current, previous, and no depression, respectively. Authors (Rude et al., 2004) found that the 1st person pronouns were mainly used in the text by currently or previously depressed participants. This is a feature of interest for the sample of participants vulnerable to depression. In our previous study (Trifu et al., 2017), we used manual scoring methodology and we found that language in depression is sensitive to the use of the first person singular pronoun and the tendency of self-focus. This is similar with the study of Zimmerman et al. (2018) who ascertained that the predominance of first-person pronoun use is associated with the severity of depression and worsening of depression symptoms in clinical inpatients. More recently, a study (Yahya and Rahim, 2023) observed a higher rate of first-person pronoun use in X (Twitter) users who exhibit depression, i.e., almost double than in average users.

The computerized analysis through LIWC in our present study shows no significant differences in the use of the 1st person singular personal pronouns between the sample with depression and controls. However, we ascertained significant differences in the use of 1st person plural personal pronouns between samples. More specifically, the self-referential and self-focused language range is active in participants with depression. These results may be explained by the specificities of Romanian language and the cultural context in which the person experiences isolation. Another explanation is that Romanian is a Romance language; this language family uses mostly non-accentuated or incomplete forms of 1st person singular pronouns; this feature is probably not detected via computerized analysis. Data from the study of LIWC- 2015 RO multilingual analysis (Dudău and Sava, 2021) support this hypothesis: “I” pronoun has the smallest usage in Romanian language with an average of 1.17 (SD = 1.12), compared with English *M* = 2.78 (SD = 2.21), and Dutch *M* = 2.81 (SD = 2.27). Brazilian Portuguese *M* = 1.87 (SD = 1.63), another Romance language, exhibited in this respect similar values with the Romanian. Moreover, Romanian language uses self-reporting (1st person singular) via inflexional and morphological verbal forms included in the conjugation, and morphemes, respectively. The subject of the sentence is often implicit in Romanian language, therefore the actual use of a personal pronoun with the verb is not necessary; this cultural feature is associated with Romanian language.

The current study examines the influence of depression and affective states on linguistic style. Other studies of language markers related to emotional states and the use of personal pronouns have differentiated between types of use of personal pronouns and their connection with affective states. For example, a study focused on language, depression and affect (Bernard et al., 2016) examines the impact of affective states on language. The result of this study suggests that both depression and temporary negative affect impact pronoun use; however, only depression influences the use of 1st person pronouns, while negative affect influences the use of 3rd person pronouns. Similarly, our study shows a lower use of impersonal pronouns in the sample of participants with depression (*M* = 2.52), compared with controls (*M* = 3.2), *p* = 0.024. As personal and impersonal pronouns are opposite types of pronouns, these results suggest the tendency of patients with depression to self-focus. This supports the theory of social disintegration (Durkheim, 2005). Moreover, the effect of 1st person personal pronouns and ‘I’-speech use in impersonal contexts appears limited, due to diminished between-persons variability (Tackman et al., 2019).

For other function words, the use of Prepositions, Auxiliary verbs, Conjunctions, and Negations categories is slightly different in the sample with depression. This sample uses less prepositions (*p* = 0.009), more auxiliary verbs (*p* < 0.001) and more conjunctions (0.010). The role of the preposition is to indicate direction, place, location, and spatial relationship (Encyclopedia Britannica, 2023; Nastachowski, 2023), or to create a connection between objects in a sentence. Limited use of prepositions appears to decrease daily communication, truncate sentences, and generate temporo-spatial disorientation. This result concurs with our previous research (Trifu et al., 2017) where linguistic markers show the use of impersonal, truncated sentences in connection with cognitive impairment. Moreover, persons with depression use short sentences, which in written language involves more use of punctuation marks, especially periods. Our results confirm this language pattern; more specifically, the use of LIWC categories All punctuation marks and Period is significantly higher in the sample with depression, compared with controls. Also, prepositions contribute to spatial cognition, together with the use of spatial terms. Limited spatial cognition and limited daily communication are characteristics of depression and other disorders based on perceptual errors, such as autism (Bochynska et al., 2020). Both people with depression, and those with autism perform poorly in the use of connective sentence structures such as prepositions. In contrast, increased use of auxiliary verbs and conjunctions in the sample of patients with depression reflects their need to focus on action and their mental state. By definition „auxiliary verbs, also known as helping verbs, are verbs used in conjunction with main verbs in order to express grammatical functions such as tense, mood, voice, aspect, and more” (IELTS, 2023). Increased use of auxiliary verbs reflects an extensive mood dysfunction, expressed through language. These data are in concordance with other observations from previous studies (Tausczik and Pennebaker, 2009). In the context of depression, people with mood changes tend to remain in a contemplative, obsessive thinking pattern and display the same revolving ideas. The presence of auxiliary verbs outlines a characteristic of this fixed pattern in thinking, while the use of strong verbs in the present tense, which express directive clear action, is limited. A longitudinal study of written samples of persons with high scores on PHQ-9, GAD-7 shows increased use of auxiliary verbs and negations in the language of people with depression and suicidal ideation (O’Dea et al., 2021). These language markers can act as robust predictors of mental health. In our study, we obtained very high scores (*M* = 5.34 compared with *M* = 3.71, *p* = 0.006) for negation (e.g., use of words like not, never, nowhere etc.). This confirms the tendency of people with depression toward negative interpretation and negative framing. Our data concur with data of another impactful study which suggests that negation words, together with negative emotions words, use of 1st person singular, use of 2nd person pronouns, and use of swear words, respectively, were “significantly positively associated with current depression symptom severity” (Kelley and Gillan, 2022, p.3).

A high number of conjunctions also emphasizes that people with depression tend to elaborate different cognitive scenarios which are very difficult to manage. When cognitive scenarios overlap, the effort to separate and clarify them generates a clutter of linguistic function words, expressing intention to communicate through language paired with poorly communicated content. Thus, patients with depression do not complete the intention to communicate through language. This increases the emotional aspect of communication, as these function words are considered primarily emotional intensifiers (Savekar et al., 2019). Their use is more automatically activated, compared with the use of the content words nouns, adjectives and verbs; hence, they occur in an increased number. The increased use of function words is evidence of a specific psychological status with diminished self-regulation and self-control, hence its potential as a linguistic marker of depression.

On a semantic level, the results of our study indicate that the use of “affect words” displays significant statistical differences. More specifically, the use of words that express positive affect is very limited in the sample with depression, compared with controls, i.e., *M* = 3.48 (2.47; 5.16) compared with *M* = 5.66 (3.99; 7.01), *p* < 0.001. This indicates that people with depression tend to lack positive attitudes – more specifically related to problem-solving, which in turn burdens everyday life. The results are similar with available literature. Newell et al. (2018) found that people with chronic stress who use depressive language also used fewer positively valenced words and more negatively valenced words. Also, Capecelatro et al. (2013) found that individuals with a history of depression longer than 5 years used fewer words related to positive emotions. A consistent observation is underlined in a study (Rude et al., 2004) that compared written language of college students who previously experienced depression symptoms. This particular group used in their language marginally fewer positive affect words and more negative affect words. In our study, negative affect words have a higher rate in the sample with depression *M* = 2.40 (1.42; 3.47) compared with controls *M* = 1.54 (1.01; 2.35), *p* = 0.011. Similar results were found in another study, where the authors underline that “negative affect predicted use of negatively focused emotion language, highlighting the potential importance of negative affect in negative emotion word use” (Bernard et al., 2016) (p.323). In our study, negative affect words were related with the presence of the anxiety valence words, *M* = 0.29 (0.00; 0.68) compared with limited or no use in controls, *p* = 0.007; however, we found no differences between samples in the use of anger and sadness affect valence words (SAW). Although the group with depression used almost double the amount of SAW *M* = 0.76 (0.14; 1.57) compared with controls, *M* = 0.38 (0.00; 1.03), the difference was not statistically significant. This contradicts expected results, and may be influenced by sample size and study methodology, i.e., the type of information requested from participants, which was to describe something nice, or pleasurable for them, not a neutral topic. Previous studies (Capecelatro et al., 2013) use the International Affective Picture System (IAPS) with more neutral stimuli. More recently, the public domain provides the Open Affective Standardized Image Set (OASIS) stimulus set, by Benedek Kurdi, Shayn Lozano, and Mahzarin R. Banaji, which proves a better choice for eliciting information from patients in future studies (Kurdi et al., 2023). Moreover, a study (Al-Mosaiwi & Johnstone, 2018) observed a lower prevalence of negative emotion and content dictionaries such as “sad,” affect,” and “feel” in a forum of people with suicidal ideation, compared with people with anxiety and depression. In this respect, is possible that the use of sadness affect words might be related with the severity of depression and the presence of suicidal ideation, which should be addressed in future studies.

Our study shows no significant differences between the sample with depression and controls for the LIWC categories that explore the cognitive and perceptual processes via language record, suggesting that both samples represent this process similarly in language. An exception is the use of tentative subcategory from the LIWC cognitive process category. The sample with depression used less tentative words *M* = 4.72 (2.83; 6.10) compared with controls *M* = 5.30 (4.27; 7.79), *p* = 0.045. This result is understandable, expected, and explainable in the context of depression. The reduced use of tentative words, i.e., that express probabilities, uncertainty, indicates patterns of absolute, black-and-white thinking, associated with radical decisions and perseverance. Previous studies (Al-Mosaiwi and Johnstone, 2018; Aguilera et al., 2019) underline the absolute thinking bias and cognitive rigidity in persons with depression.

This tendency for absolute thinking and bias in cognition also reflects in the category of Personal concerns, where the Leisure, Home and Money categories displayed statistically significant differences between the sample with depression and controls. The concern for leisure and relaxation is less important in the sample with depression *M* = 1.09 (0.59; 1.84), compared with controls, *M* = 2.21 (1.51; 3.52), *p* < 0.001; the focus of attention is the concern for the categories Home in the sample with depression, *M* = 0.55 (0.00; 0.95), compared with controls *M* = 0.22 (0.00; 0.62), *p* = 0.013, and Money, respectively, *M* = 0.27 (0.00; 0.65) in the sample with depression compared with controls, *M* = 0.00 (0.00; 0.21), *p* = 0.003. Surprisingly, we did not ascertain any difference between the depression and control samples regarding the categories of Religion and Death. Nevertheless, it is possible that these two categories become relevant within the sample of patients with depression, in specific subgroups such as those with long history of depressive symptoms, or suicidal ideation, or suicidal attempts; this may be the scope of future studies.

Likewise, the language of patients with depression indicates more focus on time components. The result of our study confirms previous results regarding verbs/ action tense and relativity category of LIWC. The language of the sample with depression is more focused on past tense *M* = 10.79 (7.83; 13.74), compared with controls *M* = 6.47 (4.61; 9.71), *p* < 0.001. This tendency of increased focus on past and diminished focus on present and future tense was also observed in our previous study (Trifu et al., 2017). The tendency is in connection with a negative exploratory style, in which the person with depression focuses on negative past events (Kellogg et al., 2020), while their capacity to project themselves in a positive present or future action diminishes (Peterson and Seligman, 1984; Pomerantz and Rose, 2014).

The use of interrogative structures underlines that people with depression tend to ask themselves and others many questions, and are more focused on asking than on finding solutions. These findings concur with the mood expression related to ruminations, which is also emphasized by the use of an increased number of past tense verbs (Eysenck et al., 2006; Groß et al., 2017). We ascertained a lower number of future tense verbs in the sample with depression than in controls, similarly with Smirnova et al. (2018). However, the use of future tense words is high in our sample with depression, which indicates an inconsistent pattern at the group level.

Body representation reflected through language is different in the sample with depression, compared with controls. Depression patients are more preoccupied with health and ingestion categories, but perform similarly with controls in the body and sexual categories. A possible explanation regarding the health category is that the group with depression extensively ruminates on different topics and actions, especially in connection with their own health, and expresses concerns on health subjects. Results regarding ingestion can be interpreted in the context of somatic symptoms associated with depression. More specifically, reduced appetite and weight loss, or increased cravings for food and weight gain are symptoms specific to depressive disorder, both in ICD-11 and DSM V (DSM, 2013).

Moreover, symptoms listed as diagnostic criteria for depression (ICD-11 and DSM V) reflect changes in drives, which in turn may be expressed through language. For example, social withdrawal as a diagnostic criterion reflects the decreased need for affiliation, which is expressed in the LIWC Drives category as decreased affiliation. In our study, the Drives category from LIWC elicits statistically significant differences between samples on multiple elements, such as affiliation, achievements and power, with higher or lower scores depending on the valence of the drives. The depression sample has lower affiliation and risk scores and higher achievements, power, and risk scores. The results confirm available literature; other studies associate depression and loneliness with lower scores for linguistic markers of social relationships and activities such as affiliation from LIWC (Liu et al., 2022). Similarly with our study, another study indicates that in the Drives category, reward words were negatively associated with depressive symptoms (Bernard et al., 2016).

These findings, taken together with experimental results of other studies, can be useful in building pre-trained language based models to augment feature-based dictionaries for programming machines to identify people at risk for or suffering from depression, since this approach has been demonstrated to be effective (Rawsthorne et al., 2020; Balagopalan et al., 2021; Malins et al., 2022; Yu et al., 2022).

## 5 Study limitations

An important limitation of this study is the relatively small sample size which might lead to classify some language parameters as irrelevant for differentiating between depressive patients and controls. The small sample size might lead to negative results concerning the influence of years of education and sex on language parameters in the regression models. Our results, however, are generally in line with studies using significantly larger sample sizes and are backed by the theoretical models generally accepted in the field. Furthermore, we did not collect information about the specific medication the patients were using, their work status, marital status and duration of illness. The effect of antidepressant medication is of particular interest in this case as some antidepressants are more “activating” and others might induce symptoms like fatigue, emotional blunting, or sedation, which might have an effect on language and thus represent an important confounding factor. Another limitation might be that we did not assess personality traits; this might act as confounding factors, since personality traits might influence both the risk for depressive disorders, and language parameters, respectively, i.e., especially through character traits like self-transcendence. To our knowledge however, there is no study published yet on the role of personality traits as a confounding factor in the relationship between the phenomenology of depression and its expression through language.

## 6 Conclusion

Depressive patients use significantly different language, in both form and content, compared with people without mood or behavioral disorders. Mainly, patients with depression use different approaches in sentence structure: short sentences, which require multiple use of the period and, implicitly, directive communication with limited content of ideas. The sample with depression predominantly use: impersonal pronouns, first person pronouns in plural form – not singular, a limited number of prepositions with increased number of conjunctions, auxiliary verbs, negations. Also, they use verbs in the past tense, much less in the present tense, an increased number of words indicating negative affects, anxiety, and a limited number of words indicating positive affects.

The main topics of interest of the sample with depression are leisure, time and money from the category Personal concerns, time from the category Relativity, and agreement from the informal language category. It is important to mention that, in our sample, the level of education acts as a predictor in the regression model, which is of interest for future research regarding the importance of the level of education as potential protective factor for depression.

## Data availability statement

The raw data supporting the conclusions of this article will be made available by the authors, without undue reservation.

## Ethics statement

The studies involving humans were approved by Ethics Commission of the Iuliu Hatieganu University of Medicine and Pharmacy in Cluj-Napoca. The studies were conducted in accordance with the local legislation and institutional requirements. The participants provided their written informed consent to participate in this study.

## Author contributions

RT: Conceptualization, Data curation, Formal analysis, Funding acquisition, Investigation, Methodology, Project administration, Resources, Software, Supervision, Validation, Visualization, Writing – original draft, Writing – review & editing. BN: Conceptualization, Data curation, Formal analysis, Methodology, Project administration, Supervision, Writing – original draft, Writing – review & editing. DH: Data curation, Investigation, Supervision, Validation, Writing – review & editing. CB-H: Conceptualization, Methodology, Resources, Supervision, Writing – review & editing. DT: Data curation, Resources, Validation, Visualization, Writing – original draft, Writing – review & editing. HC: Conceptualization, Investigation, Resources, Supervision, Validation, Writing – review & editing.

## References

[ref1] Aguilera M. Paz C. Compañ V. Medina J. C. Feixas G. (2019). Cognitive rigidity in patients with depression and fibromyalgia. Int. J. Clin. Health Psychol. 19, 160–164. doi: 10.1016/j.ijchp.2019.02.002, PMID: 31193143 PMC6517680

[ref2] Al-Mosaiwi M. Johnstone T. (2018). In an absolute state: elevated use of absolutist words is a marker specific to anxiety, depression, and suicidal ideation. Clin. Psychol. Sci. 6, 529–542. doi: 10.1177/2167702617747074, PMID: 30886766 PMC6376956

[ref3] Ansari B. Du S. (2022). Investigating user-generated content in an online Drug recovery forum: lessons for successful computer-mediated communication of social support. IEEE J. Biomed. Health Inform. 26, 5695–5703. doi: 10.1109/JBHI.2022.3196631, PMID: 35930506

[ref4] Balagopalan A. Eyre B. Robin J. Rudzicz F. Novikova J. (2021). Comparing pre-trained and feature-based models for prediction of Alzheimer’s disease based on speech. Front. Aging Neurosci. 13, 1–12. doi: 10.3389/fnagi.2021.635945, PMID: 33986655 PMC8110916

[ref5] Behdarvandirad S. Karami H. (2022). Depression, neuroticism, extraversion and pronoun use in first and foreign languages following mood induction. Lang. Sci. 94:101503. doi: 10.1016/j.langsci.2022.101503

[ref6] Berkout O. V. Cathey A. J. Berkout D. V. (2020). Inflexitext: a program assessing psychological inflexibility in unstructured verbal data. J. Contextual Behav. Sci. 18, 92–98. doi: 10.1016/j.jcbs.2020.09.002

[ref7] Bernard J. D. Baddeley J. L. Rodriguez B. F. Burke P. A. (2016). Depression, language, and affect: an examination of the influence of baseline depression and affect induction on language. J. Lang. Soc. Psychol. 35, 317–326. doi: 10.1177/0261927X15589186

[ref8] Biber D. (2004). If you look at lexical bundles in university teaching and textbooks. Appl. Linguist. 25, 371–405. doi: 10.1093/applin/25.3.371

[ref9] Bochynska A. Coventry K. R. Vulchanov V. Vulchanova M. (2020). Tell me where it is: selective difficulties in spatial language on the autism spectrum. Autism 24, 1740–1757. doi: 10.1177/1362361320921040, PMID: 32498589 PMC7545647

[ref10] Bridges K. A. Mayberg H. Van Lancker Sidtis D. Sidtis J. J. (2023). Familiar language in treatment-resistant depression: effects of deep brain stimulation of the subcallosal cingulate. J. Neurolinguistics 65:101110. doi: 10.1016/j.jneuroling.2022.101110

[ref11] Burkhardt H. A. Alexopoulos G. S. Pullmann M. D. Hull T. D. Areán P. A. Cohen T. (2021). Behavioral activation and depression symptomatology: longitudinal assessment of linguistic indicators in text-based therapy sessions. J. Med. Internet Res. 23, e28244–e28215. doi: 10.2196/28244, PMID: 34259637 PMC8319778

[ref12] Capecelatro M. R. Sacchet M. D. Hitchcock P. F. Miller S. M. Britton W. B. (2013). Major depression duration reduces appetitive word use: an elaborated verbal recall of emotional photographs. J. Psychiatr. Res. 47, 1–16. doi: 10.1016/j.jpsychires.2013.01.022.Major 23510497 PMC3732741

[ref13] Chippendale T. Gentile P. (2021). Facilitators and barriers to accepting long term care at home: an analysis of licensed home care service agency websites. Home Health Care Manag. Pract. 33, 245–249. doi: 10.1177/1084822321994779

[ref14] Chung C. K. Pennebaker J. W. (2013). Linguistic inquiry and word count (LIWC). Appl. Nat. Lang. Proc. 1999, 206–229. doi: 10.4018/978-1-60960-741-8.ch012

[ref15] Chung C. K. Pennebaker J. W. (2018). The SAGE handbook of personality and individual differences: volume I: the science of personality and individual differences. SAGE Publications Ltd. Thousand Oaks, California

[ref16] Coello-Guilarte L. Ortega-Mendoza R. M. Villaseñor-Pineda L. Montes-y-Gómez M. (2019). “Crosslingual depression detection in twitter using bilingual word alignments” in Experimental IR meets multilinguality, multimodality, and interaction. eds. Crestani F. Braschler M. Savoy J. Rauber A. Muller H. (Cham: Springer)

[ref17] DSM, (2013). Diagnostic and statistical manual of mental disorders, 5th Edition (DSM-5). American Psychiatric Association. Washington, DC

[ref18] Dudău D. P. Sava F. A. (2021). Performing multilingual analysis with linguistic inquiry and word count 2015 (LIWC2015). An equivalence study of four languages. Front. Psychol. 12, 1–18. doi: 10.3389/fpsyg.2021.570568, PMID: 34322047 PMC8311520

[ref19] Dudău D. P. Sava F. A. (2022). The development and validation of the Romanian version of linguistic inquiry and word count 2015 (Ro-LIWC2015). Curr. Psychol. 41, 3597–3614. doi: 10.1007/s12144-020-00872-4

[ref20] Durkheim E. (2005). Suicide: a study in sociology. Routledge, New York.

[ref21] Edwards T. Holtzman N. S. (2017). A meta-analysis of correlations between depression and first person singular pronoun use. J. Res. Pers. 68, 63–68. doi: 10.1016/j.jrp.2017.02.005

[ref22] Encyclopedia Britannica. (2023). Preposition. Available at:https://www.britannica.com/topic/preposition

[ref23] Eysenck M. W. Payne S. Santos R. (2006). Anxiety and depression: past, present, and future events. Cognit. Emot. 20, 274–294. doi: 10.1080/02699930500220066

[ref24] Groß J. Blank H. Bayen U. J. (2017). Hindsight Bias in depression. Clin. Psychol. Sci. 5, 771–788. doi: 10.1177/2167702617712262

[ref25] IELTS. (2023). Auxiliary verb definition and examples. Available at:https://ieltsonlinetests.com/ielts-grammar/auxiliary-verb-definition-and-examples

[ref26] Kelley S. W. Gillan C. M. (2022). Using language in social media posts to study the network dynamics of depression longitudinally. Nat. Commun. 13:870. doi: 10.1038/s41467-022-28513-3, PMID: 35169166 PMC8847554

[ref27] Kellogg R. T. Chirino C. A. Gfeller J. D. (2020). The complex role of mental time travel in depressive and anxiety disorders: an ensemble perspective. Front. Psychol. 11, 1–12. doi: 10.3389/fpsyg.2020.01465, PMID: 32848970 PMC7396699

[ref28] Kimball S. H. Hamilton T. Benear E. Baldwin J. (2019). Determining emotional tone and verbal behavior in patients with tinnitus and hyperacusis: an exploratory mixed-methods study. Am. J. Audiol. 28, 660–672. doi: 10.1044/2019_AJA-18-0136, PMID: 31430190

[ref29] Koops S. Brederoo S. G. de Boer J. N. Nadema F. G. Voppel A. E. Sommer I. E. (2023). Speech as a biomarker for depression. CNS Neurol. Disord. Drug Targets 22, 152–160. doi: 10.2174/1871527320666211213125847 34961469

[ref30] Krishnamurti T. Allen K. Hayani L. Rodriguez S. Davis A. L. (2022). Identification of maternal depression risk from natural language collected in a mobile health app. Procedia. Comput. Sci. 206, 132–140. doi: 10.1016/j.procs.2022.09.092, PMID: 36712815 PMC9879299

[ref31] Kurdi B. Lozano S. Banaji M. R. (2023). Open affective standardized image set (OASIS). Available at:https://www.benedekkurdi.com/#!portfolio/project-4.html 10.3758/s13428-016-0715-3 26907748

[ref32] Liu T. Ungar L. H. Curtis B. Sherman G. Yadeta K. Tay L.. (2022). Head versus heart: social media reveals differential language of loneliness from depression. Ment. Health Res. 1:7. doi: 10.1038/s44184-022-00014-7 PMC10955894 38609477

[ref33] Lovibond S. H. Lovibond P. F. Perţe A. Albu M. Copaciu A. (2011). DASS: manual pentru scalele de depresie, anxietate şi stres. Cluj-Napoca: Asociaţia de Ştiinţe Cognitive din România

[ref34] Malins S. Figueredo G. Jilani T. Long Y. Andrews J. Rawsthorne M.. (2022). Developing an automated assessment of in-session patient activation for psychological therapy: Codevelopment approach. JMIR Med. Inform. 10:e38168. doi: 10.2196/38168, PMID: 36346654 PMC9682451

[ref35] McDonnell M. Owen J. E. Bantum E. O. (2020). Identification of emotional expression with Cancer survivors: validation of linguistic inquiry and word count. JMIR Form. Res. 4:e18246. doi: 10.2196/18246, PMID: 33124986 PMC7665940

[ref36] Meyerhoff J. Liu T. T. Stamatis C. A. Liu T. Wang H. Meng Y. X.. (2023). Analyzing text message linguistic features: do people with depression communicate differently with their close and non-close contacts? Behav. Res. Ther. 166:104342. doi: 10.1016/j.brat.2023.104342, PMID: 37269650 PMC10330918

[ref37] Montgomery S. A. Asberg M. (1979). A new depression scale designed to be sensitive to change. Br. J. Psychiatry 134, 382–389. doi: 10.1192/bjp.134.4.382 444788

[ref38] Monzani D. Vergani L. Pizzoli S. F. M. Marton G. Pravettoni G. (2021). Emotional tone, analytical thinking, and somatosensory processes of a sample of Italian tweets during the first phases of the COVID-19 pandemic: observational study. J. Med. Internet Res. 23:e29820. doi: 10.2196/29820, PMID: 34516386 PMC8552964

[ref39] Nastachowski B. (2023). Academic guides: grammar: prepositions. Available at:https://academicguides.waldenu.edu/writingcenter/grammar/prepositions

[ref40] Newell E. E. McCoy S. K. Newman M. L. Wellman J. D. Gardner S. K. (2018). You sound so down: capturing depressed affect through depressed language. J. Lang. Soc. Psychol. 37, 451–474. doi: 10.1177/0261927X17731123

[ref41] O’Dea B. Boonstra T. W. Larsen M. E. Nguyen T. Venkatesh S. Christensen H. (2021). The relationship between linguistic expression in blog content and symptoms of depression, anxiety, and suicidal thoughts: a longitudinal study. PLoS One 16, e0251787–e0251717. doi: 10.1371/journal.pone.0251787, PMID: 34010314 PMC8133457

[ref42] Pennebaker J. W. (2023). Linguistic Inquiry and Word Count (LIWC). Available at:https://www.liwc.app/

[ref43] Pennebaker J. W. Booth R. J. Boyd R. L. Francis M. E. (2015). Linguistic inquiry and word count: LIWC2015. Available at:www.LIWC.net

[ref44] Peterson C. Seligman M. E. P. (1984). Causal explanations as a risk factor for depression. Psychol. Rev. 91, 347–374. doi: 10.1037/0033-295X.91.3.347, PMID: 6473583

[ref45] Pomerantz A. M. Rose P. (2014). Is depression the past tense of anxiety? An empirical study of the temporal distinction. Int. J. Psychol. 49, 446–452. doi: 10.1002/ijop.12050, PMID: 25355667

[ref46] Rawsthorne M. Jilani T. Andrews J. Long Y. Clos J. Malins S., (2020). ExTRA: explainable therapy-related annotations. 2nd workshop on interactive natural language technology for explainable artificial intelligence, Available at: https://aclanthology.org/2020.nl4xai-1.4

[ref47] Robertson C. Carney J. Trudell S. (2023). Language about the future on social media as a novel marker of anxiety and depression: a big-data and experimental analysis. Curr. Res. Behav. Sci. 4:100104. doi: 10.1016/j.crbeha.2023.100104, PMID: 37397228 PMC10308542

[ref48] Rude S. S. Gortner E. M. Pennebaker J. W. (2004). Language use of depressed and depression-vulnerable college students. Cognit. Emot. 18, 1121–1133. doi: 10.1080/02699930441000030

[ref49] Santos W. R. D. de Oliveira R. L. Paraboni I. (2023). SetembroBR: a social media corpus for depression and anxiety disorder prediction. Lang. Resour. Eval. 9:0123456789. doi: 10.1007/s10579-022-09633-0

[ref50] Savekar A. Tarai S. Singh M. (2019). “Linguistic markers in individuals with symptoms of depression in bi-multilingual context” in Early detection of neurological disorders using machine learning systems. eds. Paul S. Bhattacharya P. Bit A. (Hershey: Medical Information Science Reference/IGI Global)

[ref51] Sengun S. Santos J. M. Salminen J. Milenkovic M. Jansen B. J. (2023). Is death only the beginning? How people mourn artificial characters in social media. Games Cult. 11:190195. doi: 10.1177/15554120231190195

[ref52] Smirnova D. Cumming P. Sloeva E. Kuvshinova N. Romanov D. Nosachev G. (2018). Language patterns discriminate mild depression from normal sadness and euthymic state. Frontiers. Psychiatry 9:105. doi: 10.3389/fpsyt.2018.00105, PMID: 29692740 PMC5902561

[ref53] Spruit M. Verkleij S. de Schepper K. Scheepers F. (2022). Exploring language markers of mental health in psychiatric stories. Appl. Sci. 12:179. doi: 10.3390/app12042179

[ref54] Stanton A. M. Meston C. M. Boyd R. L. (2017). Sexual self-schemas in the real world: investigating the ecological validity of language-based markers of childhood sexual abuse. Cyberpsychol. Behav. Soc. Netw. 20, 382–388. doi: 10.1089/cyber.2016.0657, PMID: 28570129 PMC5510035

[ref55] Tackman A. M. Sbarra D. A. Carey A. L. Donnellan M. B. Horn A. B. Holtzman N. S.. (2019). Depression, negative emotionality, and self-referential language: A multi-lab, multi-measure, and multi-language-task research synthesis. J. Pers. Soc. Psychol. 116, 817–834. doi: 10.1037/pspp0000187 29504797

[ref56] Tausczik Y. R. Pennebaker J. W. (2009). The psychological meaning of words: LIWC and computerized text analysis methods. J. Lang. Soc. Psychol. 29, 24–54. doi: 10.1177/0261927X09351676

[ref57] Toma C. L. D’Angelo J. D. (2015). Tell-tale words: linguistic cues used to infer the expertise of online medical advice. J. Lang. Soc. Psychol. 34, 25–45. doi: 10.1177/0261927X14554484

[ref58] Trifu R. Mera D. Hambrich M. Cozman D. (2015). Verbal fluency, clustering and switching in persons with depression as indicators for cognitive impairments. Revist. Română de Psihiat. 18, 112–119.

[ref59] Trifu R. N. Nemeș B. Bodea-Hațegan C. Cozman D. (2017). Linguistic indicators of language in major depressive disorder (MDD). An evidence based research. Evid-Based Psychot. 17, 105–128. doi: 10.24193/jebp.2017.1.7

[ref60] WHO. (2022). ICD-11. ICD-11. Available at:https://icd.who.int/

[ref61] WHO. (2023). Depression. Available at:http://www.who.int/mediacentre/factsheets/fs369/en/

[ref62] Wu Y. N. Yang D. L. Jian B. A. Li C. X. Liu L. P. Li W. J.. (2023). Can emotional expressivity and writing content predict beneficial effects of expressive writing among breast cancer patients receiving chemotherapy? A secondary analysis of randomized controlled trial data from China. Psychol. Med. 53, 1527–1541. doi: 10.1017/S0033291721003111, PMID: 34425924

[ref63] Yahya N. H. Rahim H. A. (2023). Linguistic markers of depression: insights from english-language tweets before and during the COVID-19 pandemic. Lang. Health 1, 36–50. doi: 10.1016/j.laheal.2023.10.001

[ref64] Yang J. Du X. Hung J. L. Tu C. H. (2022). Analyzing online discussion data for understanding the student’s critical thinking. Data Technol. Appl. 56, 303–326. doi: 10.1108/DTA-04-2021-0088

[ref65] Yang C. Zhang X. Chen Y. Li Y. Yu S. Zhao B.. (2023). Emotion-dependent language featuring depression. J. Behav. Ther. Exp. Psychiatry 81:101883. doi: 10.1016/j.jbtep.2023.101883, PMID: 37290350

[ref66] Yu W. Zhu C. Fang Y. Yu D. Wang S. Xu Y.. (2022). Dict-BERT: enhancing language model pre-training with dictionary. Comput. Lang. 1907:150. doi: 10.18653/v1/2022.findings-acl.150

[ref67] Zhang S. Yu H. Zhang L. J. (2021). Understanding the sustainable growth of EFL students’ writing skills: differences between novice writers and expert writers in their use of lexical bundles in academic writing. Sustainability 13:5553. doi: 10.3390/su13105553

[ref68] Ziemer K. S. Korkmaz G. (2017). Using text to predict psychological and physical health: A comparison of human raters and computerized text analysis. Comput. Hum. Behav. 76, 122–127. doi: 10.1016/j.chb.2017.06.038

[ref69] Zimmerman M. Morgan T. A. Stanton K. (2018). The severity of psychiatric disorders. World Psychiatry 17, 258–275. doi: 10.1002/wps.20569, PMID: 30192110 PMC6127765

[ref70] Zinken J. Zinken K. Wilson J. C. Butler L. Skinner T. (2010). Analysis of syntax and word use to predict successful participation in guided self-help for anxiety and depression. Psychiatry Res. 179, 181–186. doi: 10.1016/j.psychres.2010.04.011, PMID: 20483481

